# A pilot protocol for conditioning music-cued relaxation responses in patients undergoing total shoulder arthroplasties

**DOI:** 10.3389/fpain.2026.1872237

**Published:** 2026-08-05

**Authors:** Janice W. Stouffer, Christina A. Myers, Susan E. Hassenbein, April D. Armstrong, Heather L. Stuckey

**Affiliations:** 1 Child Life and Integrative Therapies Program, Penn State Health Milton S. Hershey Medical Center, Hershey, PA, United States; 2 Department of Orthopaedics and Therapy Services, Penn State Health Milton S. Hershey Medical Center, Hershey, PA, United States; 3 Department of Medicine, Penn State College of Medicine, Penn State Health Milton S. Hershey Medical Center, Hershey, PA, United States

**Keywords:** anxiety reduction, autogenic relaxation, cognitive visualization, music-cued induction, music therapy, orthopedic shoulder surgery, pre- and postoperative pain management, relaxation training

## Abstract

**Background:**

Orthopedic patients undergoing shoulder arthroplasties—to treat chronic joint pain—frequently experience significant pain. Music therapy (MT) has demonstrated non-pharmaceutical benefits for reducing postoperative pain and associated perioperative anxiety. However, a study of approaches and timing is needed to enhance patient care.

**Objective:**

This qualitative study examined the effects of a conditioned MT intervention on pre/postoperative pain and anxiety among patients undergoing shoulder surgery. As part of a larger quantitative randomized controlled trial, this paper specifically evaluated qualitative participant journal comments from two music groups.

**Methods:**

Eligible adult surgery patients were consented and randomized to Live MT, Recorded MT, or Control (standard of care). Those in the two music groups received an original intervention comprising an induction song, autogenic muscle relaxation, and directed cognitive visualization over prescribed sedative guitar playing. In addition to practice recordings, a music therapist treated the Live group. Participants experienced multiple contacts with the study process, allowing for preparation, education, practice, and pre/postsurgery treatment. Journals were provided to the participants to record responses to the intervention. Comments were analyzed by the researchers and recurring themes identified.

**Results:**

From 69 MT participants, 319 comments were collected as data points. Four themes identified areas of impact: (1) The MT intervention contained qualities and components that were perceived as beneficial for relaxation and calming. (2) Participants personalized their visualization experience with the music, which cued memories and associations of soothing or reflection. (3) Music therapists were a supportive presence, in addition to the healthcare team. (4) Through practice, aspects of the music intervention became internalized as a self-help tool. Theme 1, referencing general relaxation, was further divided into three subthemes—(1a) pain management, (1b) anxiety management, and (1c) sleep hygiene—which were found to be interrelated. Pre/postsurgery comparisons were made between the Live and Recorded groups.

**Conclusions:**

Given opportunities for repeated practice, the MT intervention was effective in supporting relaxation. The effects included improved pain and anxiety management, as well as increased sleep and personalized patient experience.

**Clinical Trial Registration:**

ClinicalTrials.gov, identifier NCT02692768.

## Introduction

Orthopedic patients increasingly undergo shoulder arthroplasties to treat their immobility and pain arising from chronic joint issues (1). Shoulder arthroplasty, considered to be a painful surgical experience, requires optimization of pain management to ease restoration of mobility (2, 3). Healthcare professionals recognize that patient perceptions of pain are impacted by psychosocial risk factors such as stress and anxiety (4). Music therapy (MT) has gained attention as an avenue for relief for orthopedic patients (5, 6), providing non-pharmaceutical benefits for reducing postoperative pain and perioperative anxiety (7–9). However, most studies use commercially recorded music as the intervention, which is conducted immediately prior to surgery and/or soon after surgery, without offering opportunities for advanced practice and conditioning. Therefore, a study of MT approaches and timing of interventions is needed to fully optimize patient care.

MT has been documented as an effective modality for addressing both pain and anxiety in a multitude of populations (10, 11); however, research studying the combined effects of music listening and muscle relaxation training is limited (12). Studies focused on surgery patients report decreased pain scores, decreased anxiety, and increased tolerance for procedures (13–22). In addition, Tan et al. (23) highlight the role of anticipatory anxiety in respect to pain and surgery. The use of conditioning and relaxation training for pain and anxiety reduction is promoted by the National Institutes of Health (24) and supported by reviews of scientific literature on pain reduction specifically for hospitalized patients (25). Chi and Young (20) emphasize that repetitive music exposure increases listener comfort and heightens the relaxation experience. At present, there are only a few studies that have analyzed the benefits of introducing MT to patients well in advance of their surgical procedure. Some have explored music-based treatment beginning from the night before surgery through anesthesia and recovery, along with the use of live MT in recovery (21).

Multiple factors contribute to individual human responses to music and to the components of music that influence stress reduction (26). However, the properties of music generally accepted as appropriate to promote relaxation and sedation are documented in the literature (23, 27, 28). Music should reflect a tempo of 60–72 beats per minute. Dynamic levels should be consistent and remain within a soft-to-moderate volume range. Rhythms should be regular, without sudden fluctuations in tempo. Melodic lines are most effective when pitches are low rather than high. The meter should be based on three beats per measure, which is inherently lulling. Finally, music selection should reflect consonant, not discordant, harmonies with a soft tone quality on strings, voice, or piano. Adding verbal suggestions from the music therapist for focused breathing, along with slow, sedative music, facilitates muscle relaxation and decreased blood pressure, as evidenced in a study of hypertensive patients who participated in daily slow music–guided breathing sessions (29).

Given the logistics of clinical surgery schedules, preparatory and recovery spaces, and limited access of many medical centers to board-certified music therapists, the use of recorded music for patient relaxation may be deemed necessary at times. In healthcare, a spectrum of music applications exists, ranging from live MT interventions by board-certified professionals to the use of commercially recorded generic music played at a patient's bedside by healthcare staff (30, 31). Dileo (32) describes generic recorded music played at the bedside or while performing a procedure as “music medicine”. Conversely, she defines medical MT as being based in a therapeutic process between a board-certified music therapist and the patient, using individualized music to achieve specific medical goals.

Mixed results have been reported for the use of passive listening to commercially recorded music for medical relaxation, even when patient preference is considered (33). However, increased treatment effectiveness has been reported in studies using live, sedative music with therapeutic suggestions, given the need for immediate, patient-centered adjustment of the treatment content and the role that relationship plays in decreasing anxiety (21, 33, 34). To address both approaches, the present study used two treatment arms: one with a recorded intervention alone and the other adding live intervention at designated points surrounding surgery. With attention to the properties of the intervention, advanced timing for practice, and individualization of patient experience, utilizing MT in the pre- and postoperative period may increase the effectiveness of multimodal approaches for perioperative pain and anxiety management, with minimal, if any, negative side effects for patients.

## Materials and methods

The purpose of this non-blinded randomized study was to determine the effects of a conditioned MT intervention on pre- and postoperative pain and anxiety among patients undergoing shoulder surgery. A larger study was conducted using quantitative data to measure pain and anxiety scores, vital signs, and morphine equivalent use, comparing the MT intervention groups with patients who received standard of care without MT (35). The objective of this paper was to evaluate qualitative journal comments recorded by participants in the music groups to describe their perceptions of effect and benefit.

## Participants

Eligible participants were adults aged 18 years and older undergoing elective total or reverse total shoulder arthroplasty, with a body weight of 50–125 kg, and deemed American Society of Anesthesiologists physical classification scale 1–3. Excluded participants were those outside the specified weight range, those with non-elective surgeries, pregnant patients, non-English-speaking patients, those with pre-existing hearing problems, those diagnosed with cognitive disorder, including psychosis and dementia and/or musicogenic epilepsy, and patients with anatomical abnormalities of the shoulder, such as cancerous lesions or congenital defects.

### Recruitment, consent, and randomization

Participants were prospectively identified from a list of scheduled elective shoulder surgeries performed by the study investigator at a single institution. Those meeting the inclusion criteria were contacted by telephone utilizing a script to ascertain their interest in participating in the study. Interested participants met with the research team at the time of the preoperative appointment, where a research team member explained the study and obtained informed consent according to the institution's Human Subjects Protection Board guidelines (STUDY00004093). No compensation for participation was provided. A sealed envelope approach was used to randomize participants into one of three treatment allocations: Live MT; Recorded MT; or Control (No MT). The envelopes were randomly sorted.

### Materials

The materials included a six-string acoustic guitar and an iPad for intervention lead sheets stored in the OnSong mobile application (36). Music lead sheets from a variety of genres were available in case the therapist's clinical judgment indicated a need for adapted interventions for the live group. This included music that was potentially familiar and preferred by a participant for extended calming. Recordable CDs and CD players were determined best for this study, given that thumb drives and mp3 links were not considered most readily accessible to many participants. A Participant Journal was provided to participants in the music groups to record observations and comments regarding their experience with the MT intervention. The control group received no additional counseling or intervention to which they could respond; therefore, they were not assigned journals.

### Intervention rationale

Pre- and postsurgery music interventions for the current study were provided in the Same Day Unit (SDU) and the Post-Anesthesia Care Unit (PACU), both of which are open environments and receive patients at various stages in their preparation and recovery. Therefore, as compared to live music-making techniques for pain management, an intervention that presented music volume in a controlled manner was deemed necessary at the time of study design. Based on evidence-based literature and clinical practice experience with hospital patients, the intervention consisted of three phases: induction song, autogenic muscle relaxation, and directed cognitive visualization. Prescribed sedative guitar (arpeggiated guitar playing in three-quarter time at 60 bpm with one note per beat for entrainment) was maintained throughout the 30-min intervention, using musical phrasing and cadences for transition and closure of verbal content. Consistent with medical pain and anxiety management literature (30, 37), the intervention was designed as a receptive approach, in which patients were not expected to actively participate in making music, verbally contribute to the visualization scenes during presentation, nor engage in verbal processing with the therapist. The receptive approach allowed for variation in states of wakefulness within each patient, encouraging rest and progression to sleep when possible.

## Music induction

Relaxation protocols often emphasize the need for transitioning from current activity and state of mind into a mental space that will be receptive and attentive to suggestion. The study's induction elements were composed in simple musical form and sedative style to facilitate a successful transition into the relaxation experience. Four short verses invited the participant to focus on a different aspect of their mind–body connection, beginning with simply listening and attending to the therapist's guitar playing and voice, thus gradually bringing the attention inward. Subsequent verses added focused breathing, progressed to suggestion of global muscle relaxation, and culminated in calming the mind as a final release of active thoughts. A sedative guitar interlude was played between verses, allowing time for response and building mindfulness (see Supplementary Appendix A for details).

## Autogenic versus progressive muscle relaxation

Muscle relaxation therapy reduces the activity of the sympathetic nervous system, resulting in lower heart rate, respiratory rate, and blood pressure. The process assists in regulation of peripheral and central nervous systems, thereby decreasing stress, anxiety, and depression and assisting in the management of different health problems (38). Relaxation influences pain by decreasing the tissue oxygen requirement and lowering lactic acid. In turn, this relieves skeletal muscle tension and increases endorphins, improving symptoms of anxiety. Kwekkeboom et al. (39) explored the variables of progressive muscle relaxation (PMR) and analgesic imagery interventions in cancer pain. Of interest was the investigators’ report that patients participating in the PMR group were instructed not to tense muscle groups that were strained or that increased pain. In contrast, autogenic training (AT) is a relaxation technique commonly used in multimodal pain management. AT utilizes mental concentration to impact the person's physical condition and directs their attention to relaxation reactions in the body (40). As compared to PMR, AT does not require the patient to create additional muscle tension in the painful muscle areas or, particularly in the hospital setting, body areas affected by surgery sites, IVs, chest tubes, or more. In the present study, participants were invited to methodically pay attention to their bodies from head to toe, following suggestions for releasing tension in each area while supported by sedative guitar playing (see Supplementary Appendix B for details).

## Visualization scripts

Directed cognitive visualization, widely considered a form of mind–body treatment, uses imagination and mental imagery to focus awareness and promote relaxation. Facilitators usually lead participants through calming images based on multiple senses for the visualization to be vivid. Nguyen and Brymer (41) studied the mental health effects of guided imagery on state anxiety, noting that nature-based suggestions achieved a greater reduction in anxiety than urban-based suggestions. In addition, embedding metaphors and affirmations into guided imagery scripts may be effective in promoting personal coping and healing potential (42).

Drawing on previous clinical experience, these MT investigators identified the following scenes as frequently chosen for personal visualization with music: the beach, a forest stream, and a meadow. Standardized scripts were created to describe each according to the senses, while maintaining an open flowing quality to match the lulling aspect of the three-quarter-time sedative guitar accompaniment. Further details and metaphors infused each scene with qualities of strength, peacefulness, and hope, which then became suggested affirmations for the participant. Repeated suggestions for smooth, even breathing were included. The scripts ended with suggestions for either continued deep rest or transition of awareness back to the current environment, with therapists using clinical judgment for the live sessions, based on the patient's degree of sleep or awareness (see Supplementary Appendices C–E for details).

Thus, a novel intervention protocol was developed to include music-based induction, autogenic muscle relaxation, and cognitive visualization of nature scenes imbued with the qualities of strength and affirmation. The role of patient preference was recognized by inviting participants to choose one of the three scenarios for their guided visualization. Those randomized to the music treatment groups had contact with the study process at specific points in their surgery schedule to allow for preparation and education, practice and repetition, anxiety reduction before surgery, and pain management following surgery.

As shown in Table 1, interventions were performed and data collected for each group across six different perioperative phases of care, including preoperative, presurgery (SDU), postoperative (PACU), and 12–24 h postoperative (inpatient). Not all quantitative measure time points are included in Table 1; additional details are reported in Kim et al. (35).

**Table 1:** Process and design.

Study steps	Preoperative	Presurgery (SDU)	Postsurgery (PACU)	Recovery (inpatient floor)	2 weeks postop	6 months postop
Consent and randomization	L,R,C					
MT intervention	L,R	L,R	L,R	L,R		
APP 4—participant journal	L,R				L,R	
APP 3A—recording use				L,R		
APP 9—patient follow-up survey						L,R

L, live; R, recorded; C, control.

### Preoperative visit

Participants randomized to the Live and Recorded MT groups were met by the music therapist. An overview of the treatment intervention was provided, and each participant was given the opportunity to indicate preference of one guided visualization scenario to be included in their 30-min music relaxation routine. Their choice was recorded on the MT Patient Demographic Sheet (Supplementary Appendix F). The Live MT group engaged with the live MT intervention as described previously. Following the live intervention, participants were given a recorded CD of the same content for home practice before surgery. For safety, and based on the understanding that many patients learn to guard their shoulder position to minimize pain, the participants were counseled to use a pillow or towel roll under their affected shoulder while listening to the intervention. This might support a more comfortable position after potential relaxation of the muscles. The participants were also given a journal to record the frequency of practice prior to surgery and open-ended personal responses or comments.

The Recorded MT group received the same intervention overview and the same choices for the cognitive visualization scenario. Participants were given a prerecorded CD to take home corresponding to their scenario, consisting of the same 30-min treatment as the Live group, but with no live intervention. Safety counseling for use of a pillow or towel roll was provided. The participants were given a journal, with the same instructions for use as the Live MT group. The control group did not receive any type of music therapy intervention, nor were they given a journal.

### Presurgery visit

Prior to being transferred to the operating room, the Live MT group participants received a live presentation of their selected music-assisted relaxation session. The music therapist used clinical judgment to adapt the pace and content of the intervention, in response to any interruptions or participant response. The Recorded MT group participants were given a CD player and a CD with their recorded music protocol. The recording was initiated, and the therapist left the room. The control group received standard of care without MT intervention.

### Postsurgery, post-anesthesia care unit

Following surgery, all participants were transported to the PACU. As per standard of care, nursing staff set up a hydromorphone patient-controlled analgesia pump with standard dosing for each patient. The Live MT group received the same live intervention as presurgery, along with reminders for focused breathing and any clinically indicated adaptations. A CD player was given, with suggestions to the participants and nursing staff to use the recording as needed while in the PACU. Participants in the Recorded MT group listened to their recorded music intervention via the CD player, which was set up by the music therapist, who then left the room. Participants in both MT groups were encouraged to continue using the relaxation CD at least once every four hours after transferring out of the PACU to the acute care floor, unless sleeping. The control group received standard of care without MT intervention.

### Inpatient 12–24 hours after surgery

At 12–24 hours after surgery, the Live MT group received the same live intervention as previously. The Recorded MT group participants were offered the option to listen to their recording one additional time. Both music groups were reminded to continue using their music-assisted relaxation CDs at home and to document their responses in their journals.

### 2-Week postop visit

At approximately 2 weeks postoperatively, total shoulder arthroplasty patients routinely returned to the clinic for medical follow-up. Both music groups completed an MT Patient Evaluation Survey. Both groups were reminded to continue using their music-assisted relaxation CDs at home and to document their responses to the study in their journals.

### 6-Month postop visit

At 6 months postoperatively, patients routinely returned to the clinic for medical follow-up. Journals were collected from the participants in both music groups.

## Data collection and analysis

Participants in both MT groups were given a Participant Journal to take home (Supplementary Appendix G). The journal was intended to provide for the recording of open-ended responses or comments, with each participant specifically invited to share “any and all” feedback regarding their study experience, the intervention, and the process. Study data were collected and managed using REDCap electronic data capture tools hosted at the Penn State Health Milton S. Hershey Medical Center and Penn State College of Medicine (43). Comments from the two MT groups were analyzed both separately and together using a qualitative content analysis (44, 45). Three researchers took notes on frequently recurring words or phrases and then coded the data using inductive means rather than applying an *a priori* theory. The initial seven codes were discussed among the team, with the qualitative researcher serving as the arbitrator, and a storyline was created. An audit email trail of coding decisions was maintained. From there, four initial themes were developed and revised four times with the group before being finalized by the group. Subsequently, the codes were matched to the themes. Frequently, participants referenced multiple codes in the same comment. Therefore, for each code, reporting of data included the number of individual participants who referenced that code and the number of total comments linked to that code by all participants in aggregate. Within the limits of this pilot study population, the data confirmed existing categories and indicated thematic saturation.

## Results

A total of 122 people (*n* = 122) were enrolled in the study, with 14 withdrawals. Participant demographics are outlined in Table 2. Of the 69 participants who were randomized to the MT Live and Recorded groups combined, 54 submitted journals, amounting to a 78% rate of return. Forty-nine journals contained written comments: 24 from Live MT and 25 from Recorded MT. The remaining five journals contained dates of CD use but no comments. Use of the journal in regard to demographics, timing, and treatment group is presented in Table 3. A total of 319 comments were contributed as data points: 154 from Live MT and 165 from Recorded MT. Each comment referenced one or multiple codes as data were being analyzed; therefore, some comments were tallied under more than one code. For example, “relaxed and slept” was journaled as one comment but was tallied under both the Relaxation and the Sleep Hygiene codes, given that it referenced both. Six of the 319 comments, contributed by four people, were not clear or informative enough to be coded.

**Table 2:** Participant demographics.

Demographic	Years/percent of participants
Mean age (years)	67.59 (9.38 Sample SD)
Sex—male	21 (38%)
Sex—female	33 (61%)
Race—Caucasian	54 (100%)
Surgery type—total TSA	35 (64%)
Surgery type—reverse	19 (35%)

TSA, total shoulder arthroplasty.

**Table 3:** Record of participant journal use.

Demographics and timing	Recorded MT group	Live MT group
Number of journals returned containing comments	25	24
Male	11	11
Female	14	13
Number of journals used presurgery	24	22
Male	10	11
Female	14	11
Number of journals used postsurgery	15	13
Male	5	7
Female	10	6
Number of journals used both pre/post	16	12
Male	6	7
Female	10	5

Following an independent review of journal comments by three study investigators, responses were categorized as either having effect or neutral/no effect. From this broad foundation for interpretation, participant perceptions of the intervention and study process were evaluated. At some point in their timeline, most of the Live and Recorded MT participants used words such as “helpful,” “positive result,” and “better” to describe their reactions (314 comments). Notations (5) such as “no benefit,” “got tired of doing it,” and “taking time from other activities I prefer to be doing” were considered neutral or indicating no effect, as represented by five participants (four LMT, one RMT). In regard to patient safety, no adverse effects were reported by study participants, either in journals or by verbal report. One person very early in the study reported “increased pain with music because it relaxes (the shoulder) into a position that puts him in pain.” Thereafter, all participants were counseled to prop their shoulder prior to the intervention, as noted in the study procedures for the preoperative visit.

From the responses indicating perception of effect, seven codes were consistently referenced: (1) relaxation and calming, (2) pain management, (3) anxiety management, (4) sleep hygiene, (5) cognitive associations, (6) patient experience, and (7) internalization. These codes were developed into four themes that identified areas of impact on the participants. Given relationships that emerged between codes, Theme 1, which referenced general calming and relaxation, was further divided into three subthemes: (1a) pain management, (1b) anxiety management, and (1c) sleep hygiene. All definitions are provided in Table 4. In addition to codes for the main themes, one code for Music and one for Imagery were developed to capture participant comments related to the intervention.

**Table 4:** Themes and definitions of codes.

Theme	Code	Definition	No. of comments/percent of total comments	No. of people/percent of participants						
					Live group			Recorded group		
				Total people	Total live	Presurgery	Postsurgery	Total recorded	Presurgery	Postsurgery
**Theme 1:** The MT intervention contained qualities and components that were perceived as beneficial for relaxation and calming	Relaxation and calming	Perception of feeling calm, relaxed, restful, or decreased muscle tension	209/65.5%	47/96%	23/95.8%	22/91.6%	12/50%	24/96%	22/88%	15/60%
** Subtheme 1a:** Pain management	Pain	Perception of physical pain and discomfort	52/16.3%	20/40.8%	10/41.6%	6/25%	6/25%	10/40%	5/20%	7/28%
** Subtheme 1b:** Anxiety management	Anxiety	Perception of emotional feelings of decreased anxiousness, decreased racing thoughts, and peacefulness	21/6.26%	21/42.8%	14/58.3%	11/45.8%	3/12.5%	7/28%	7/28%	0
** Subtheme 1c:** Sleep hygiene	Sleep	Effect on ability to fall asleep, duration, and/or quality of sleep	113/35.4%	34/69.3%	15/62.5%	14/58.3%	8/33.3%	19/76%	19/76%	11/44%
**Theme 2:** Participants personalized their visualization experience, which cued a memory and associations of soothing or reflection	Cognitive associations	Thoughts evoked by the music or cognitive visualization scripts, which brought memories of previous experiences, interactions, or connections to imagined experiences	13/4%	7/14.2%	5/20.8%	4/16.6%	1/4.16%	2/8%	2/8%	0
**Theme 3:** Music therapists were a supportive presence, in addition to the healthcare team	Patient experience	Interaction with the healthcare system, aspects of their care, and the perception of attention and communication	14/4.38%	7/14.2%	4/16.6%	2/8.3%	2/8.3%	3/12%	2/8%	1/4%
**Theme 4:** Through practice, aspects of the music intervention became internalized as a self-help tool	Internalization	Purposeful practice of the intervention, familiarity, memorization and use, and automatic responses	17/5.33%	9/18.3%	4/16.6%	3/12.5%	1/4.16%	5/20%	4/16%	1/4%
	Imagery	References to image, visualization, and scene	4/1.25%	4/8.16%	2/8.3%	2/8.3%	0	2/8%	1/4%	1/4%
	Music	References to music, guitar, singing, and lyrics	25/7.83%	18/36.7%	13/54%	11/44%	3/12.5%	5/20%	3/12%	2/8%
	Neutral/no effect	References to no effect or benefit; statements not clearly positive or negative	5/1.56%	5/10.2%	4/16.6%	3/12.5%	1/4.16%	1/4%	1/4%	0
	Unable to code	Not clear; no informative reference to the study	6/1.88%	4/8.16%	1/4.16%	1/4.16%	0	3/12%	2/8%	1/4%

### Theme 1: the MT intervention contained qualities and components that were perceived as beneficial for relaxation and calming

A total of 47 participants referenced perceptions of relaxation or calming effects as a result of the intervention (209 comments). Using words such as “restful,” “soothing,” “calming,” and “totally relaxed,” they communicated awareness of a positive state. Although some were at first “amused” to have the music and music therapists in the room, the intervention subsequently helped them relax. Some of the representative comments were as follows:

With pain having been manageable, I have used this meditation frequently to simply relax.

Beautiful soothing voice.

After I started following the lyric/instructions, I found the process very relaxing.

Music is peaceful.

Able to relax to the CD.

In certain instances, participants perceived being so relaxed that they felt separated from their bodies. It was described as being close to a “spiritual experience” for some:

Started to relax and feel like floating away.

Felt totally relaxed—when opened my eyes, the rest of my body stayed so relaxed it was like it wasn't connected.

Perceptions regarding this general relaxation effect were noted by 23 people in the Live MT group (22 presurgery, 12 postsurgery) and 24 in the Recorded MT group (22 presurgery, 15 postsurgery). Some patients reported in both the pre and postsurgery time periods; therefore, figures given in the text do not tally with the ones given in the brackets.

#### Subtheme 1a: pain management

References to pain were made by 20 participants, noting relief from pain or improved perception of pain (52 comments). A few comments specified improved pain alone:

Off Oxy pain meds; hurt; CD helped Post PT. Some relief 1–2 degrees.

Today I began PT and the pain is strong but meditation allowed it to be tolerable.

Frequently, however, reports of increased pain management were interwoven with a general relaxation effect:

When pain level increased and discomfort from constipation, listened to CD with a positive result in pain reduction and calming.

High level pain, took extensive time to relax, after hour or more of music, pain was less.

Woke tense and hurting a lot. Definitely calmed me. Much better.

Shoulder was really hurting even after pain meds. CD relaxed and reduced pain level.

Other comments pointed to the participants actively using the music intervention to shift their minds to a different state, going as far as stating the music helped put them “above the pain” or allowed them “to forget the pain for a while.” One participant verbally commented that music was most beneficial right after surgery (PACU) as it helped “distract” her from pain:

Shoulder was very sore from surgery. The tape was a good distraction and allowed me to forget the pain for a while.

No pain when music was playing. Put mind above the pain. I knew pain was there but you went above it. Pain wasn't the focal point.

These statements suggested that the music intervention helped the participants actively shift their attention. Thus, whether through general relaxation or active shift of attention, the participants experienced positive changes in pain control.

The participants also associated increased pain control with ease of sleep, weaving these themes together. Of these comments, not all reported a decrease in or absence of pain while using the music intervention. However, these participants perceived the music intervention to increase their ability to manage pain or redirect their thoughts enough to promote sleep:

Music helped relax me enough to put me to sleep which resulted in decreased pain because I could sleep.

Used to transition to sleep; helped with pain.

Started cutting back on pain pills (oxycodone) today so falling asleep was not as easy as it had been earlier in the week. After lying in bed for an hour, I decided to try the CD. It took until nearly the end of the program but I did finally relax enough to fall asleep.

The positive impact of the music-based relaxation improved manageability of pain, and facilitated sleep. Comments related to pain were made by 10 participants in the Live MT group (six presurgery, six postsurgery) and 10 in the Recorded MT group (five presurgery, seven postsurgery). Some patients reported in both the pre- and postsurgery time periods; therefore figures given in the text do not tally with the ones given in the brackets.

#### Subtheme 1b: anxiety management

References to anxiety were made by 21 participants (21 comments). Using words such as “anxious,” “stressed,” and “slow my mind,” they communicated awareness of a shift in state:

As increasing apprehension begins, I was able to return to relaxed focus.

Helped me more with anxiety vs. pain—music helped calm me down and stop thinking about what I had on my mind

Music helped with anxiety, especially when I felt “out of my mind.”

Participant statements suggested that they experienced an improved state after slowing down their mind and body as a result of listening to the sedative music and following the sung and verbal cues.

Similar to participants linking pain management and sleep, some also linked anxiety management and pain management:

Woke with migraine and increased pain. Took meds and listened to CD. Felt music was helpful in relieving stress from migraine and relaxing pain.

Relaxation helped with stress—pain still present but pushed aside.

Comments related to anxiety were made by 14 participants in the Live MT group (11 presurgery, three postsurgery) and seven in the Recorded MT group (seven presurgery, 0 postsurgery).

#### Subtheme 1c: sleep hygiene

A total of 34 participants documented use of the recordings as preparation for sleep or as resulting in increased ease of sleep (113 comments). After listening to the practice CD, several participants noted “fell asleep while listening” prior to the end of the recording, indicating a relaxing and calming effect:

Very relaxing, fell asleep before completion.

I found that listening to tape before sleep allowed for a more restful sleep.

Relaxation led to sound sleep, only up two times.

Again, a sleepless night, was able to relax and sleep.

The emerging relationship between feelings of calm and improved ability to sleep was exemplified in multiple comments, which reported being better able to sleep specifically because of the music intervention prompting a decrease in stress. Sleep references such as “it helped me wind down and relax for sleep” were associated with navigating their anxiousness:

Relaxed, positively affected stress/anxiety. Eventually fell asleep.

Mind still wandering then became sleepy and fell asleep before tape was over.

Other sleep references were associated with pain control. The participants linked their relaxation response and pain control to increased ability to sleep:

After being jolted awake by rolling onto my injured shoulder, I started listening to CD and was able to relax and return to sleep.

The participants experienced the intervention as calming to the point of reducing their stress. For some, the feeling of increased relaxation led to less pain and better sleep. Notations were timed such that they described effects both during the days awaiting surgery and following surgery during the recovery period (15 participants in the Live MT group: 14 presurgery, eight postsurgery; and 19 participants in the Recorded MT group: 19 presurgery, 11 postsurgery). Some patients reported in both the pre- and postsurgery time periods; therefore figures given in the text do not tally with the ones given in the brackets.

### Theme 2: participants personalized their visualization experience with the music, which cued memories and associations of soothing or reflection

Notations made by seven participants (13 comments) related to cognitive association or thoughts evoked by the music or cognitive visualization scripts. Some comments reflected memories of previous experiences or interactions, while others were connected to vivid scenes in their imagination:

Took me back to nursery rhymes as a child—very nice.

Cried at first because music reminded me of someone who used to play similar music at my restaurant in AK back in 2006. I then started to relax body areas.

Tried to imagine sitting on the beach reading.

Aspects of participants personalizing their own experience took several forms, including remembering a certain associated experience, or changing their visualization from what was cued by the intervention to what was more reflective of their life experiences:

I thought that when in forest sleeping that I wouldn't be able to see sky & clouds because of overarching tree branches so I imagined being in a clearing among the trees. In other words, the script wasn't true to life as I knew it but then I mentally changed the scene.

These associations were perceived by the patients as improving their state of relaxation and their treatment experience. Comments were contributed by five participants in the Live MT group (four presurgery, one postsurgery) and two in the Recorded MT group (two presurgery, 0 postsurgery).

### Theme 3: music therapists were a supportive presence, in addition to the healthcare team

Seven participants referenced the music therapist in their comments (14). These notations reflected that the music therapist’s presence was viewed as supportive and contributed positively to the participants' ability to feel calm and cope. The music therapy intervention was personalized, which provided increased comfort and fostered feelings of connection with providers. By having a dedicated therapist present, the participants reported feeling like the music guided them to “a safe space”:

The live music in preop and just before discharge helped greatly.

Live music provided a nice personal touch—I felt like I won the lottery.

Message seemed directed specifically to me. Kind and calming. Awake while listening.

I began to think how wonderful it was to have this personal recording made for me.

These comments referred to the personalization that was offered to the participants by their choosing one of the three nature scenes they desired for the cognitive visualization component. The comments also alluded to patients having an increased sense of the music therapist being “there with me.” References to the music therapist or patient experience were made by four participants in the Live MT group (two presurgery, two postsurgery) and three in the Recorded MT group (two presurgery, one postsurgery).

### Theme 4: through practice, aspects of the music intervention became internalized as a self-help tool

Nine participants practiced listening to the music intervention frequently (17 comments). They shared that they were “humming the music” to themselves or “singing” the song to themselves between uses of the recordings. “Using catchphrases from the CD at times when I'm uncomfortable” indicated having used the recording to the point of remembering the music cues for breathing and relaxation:

I've learned the induction song and sing it to myself.

The music has helped me learn to block things out of my mind and relax.

I've memorized the song and I find I often sing it to myself when I'm stressed.

Having the intervention recordings to use in the days prior to surgery was beneficial from the standpoint of practice and familiarity. This “familiarity” and internalization of music cues helped some “feel prepared to relax” on the day of surgery:

Seems automatic—as soon as the music starts, my mind and body relax.

Fell asleep thinking about how this recording will be familiar and soothing when I'm in the hospital pre-op and post-op.

These comments point to the potential benefits of music and guided intervention becoming a trigger, or cue, for the development of automatic responses. The participants internalized the intervention as a self-help tool. References to internalization were made by four participants in the Live MT group (three presurgery, one postsurgery) and five in the Recorded MT group (four presurgery, one postsurgery).

With regard to the components of the intervention, some participants commented on the sedative quality of the intervention with entries such as “repetitiveness is relaxing” and “hypnotic.” A few generally “enjoyed the music and images.” Eighteen participants specifically referenced the music and “lyrics” (25 comments). Some focused on the music of the guitar and singing. Many participants shared that the “low soothing tones” helped them relax “inwardly and outwardly”:

I'm amazed how the music can put your mind at ease.

Favorite is singing, so soft and sweet.

Felt the melody take over my mind.

Words are distracting, I just listen to the music.

These comments were made by the following participants: 13 in the Live MT group (11 presurgery, three postsurgery) and five in the Recorded MT group (three presurgery, two postsurgery). Some patients reported in both the pre- and postsurgery time periods; therefore figures given in the text do not tally with the ones given in the brackets. Several participants (4) made note of the guided visualization itself:

I thought that when in forest sleeping that I wouldn't be able to see sky & clouds because of overarching tree branches so I imagined being in a clearing among the trees. In other words, the script wasn't true to life as I knew it but then I mentally changed the scene.

Calming—breathing—beach a great place to direct thinking.

Relaxing, narration helpful.

Calming message very nice.

Imagery references were made by the following participants: two in the Live MT group (two presurgery, 0 postsurgery) and two in the Recorded MT group (one presurgery, one postsurgery). For some, including more of a song-based approach would have been helpful, rather than transitioning to verbal guidance over sedative guitar. One person expressed that the “music was a bit slow.” Other suggestions regarding intervention adaptations were shared as well:

Relaxed. I would prefer hearing the ocean instead of guitar music. I would not be distracted by the strumming of the guitar.

I would very much like to hear real ocean sounds under the guitar music or add music at the end after the guided phase is finished???

Certain participants used the recording multiple times in one sitting, either because they received a positive effect and wanted that effect to continue or because they needed a longer duration of the intervention to achieve a positive effect. Several participants randomized to the Live MT intervention journaled statements indicating motivation to extend the use of the CD at home within one sitting, listening multiple times on repeat:

Night before surgery—First time helped me relax my body but mind was still racing—second time through (about an hour later), I relaxed enough to finally fall asleep.

Wife and I listened several times (4) before going to bed to concentrate on wording—listened last time in bed—very relaxing night before surgery.

Pain level high but after listening 3 times felt a mellow feeling ok; music was benefit by 7:00.

**Figure 1:**
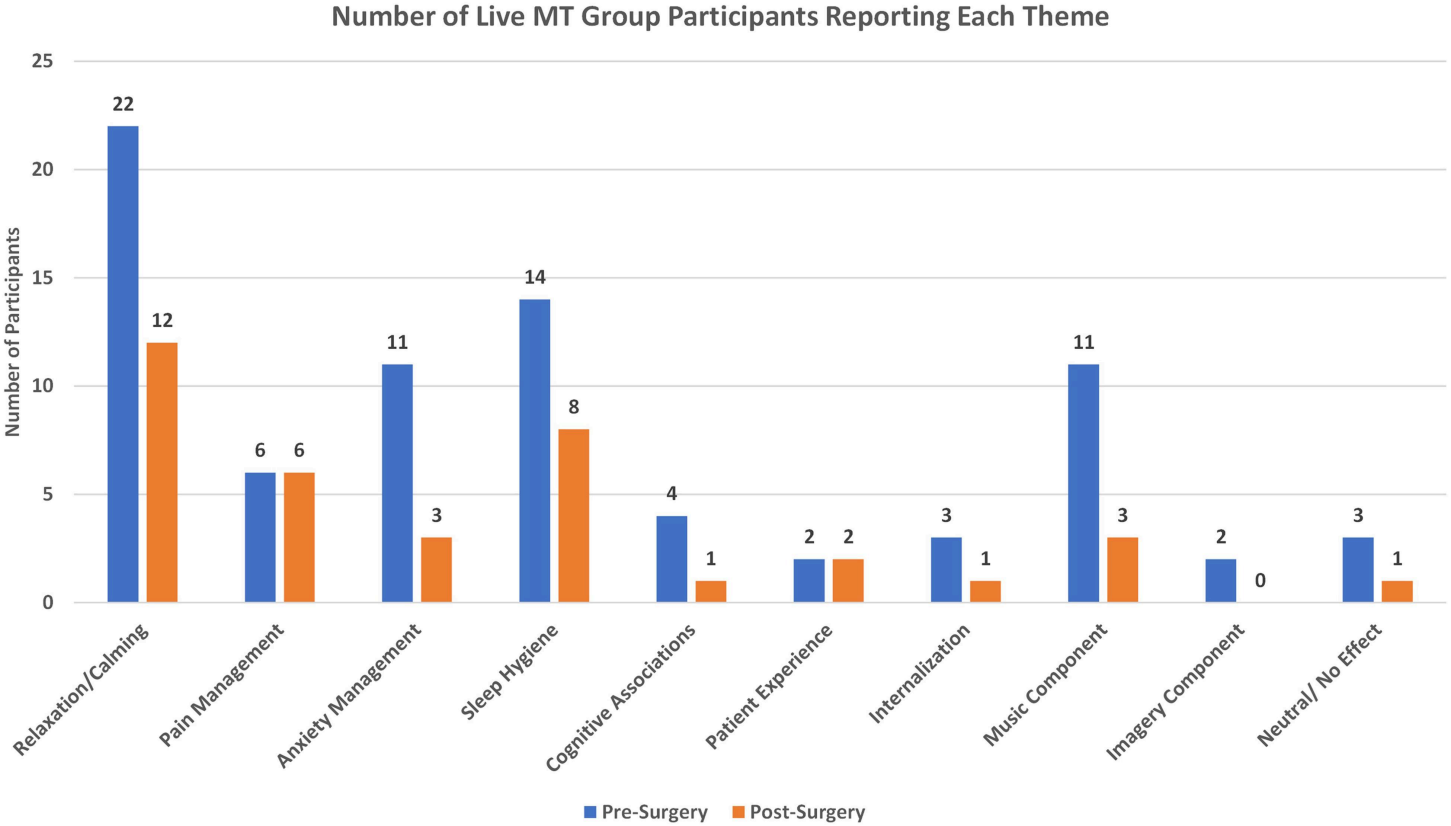
Live MT group: pre/postsurgery comparison of themes.

**Figure 2:**
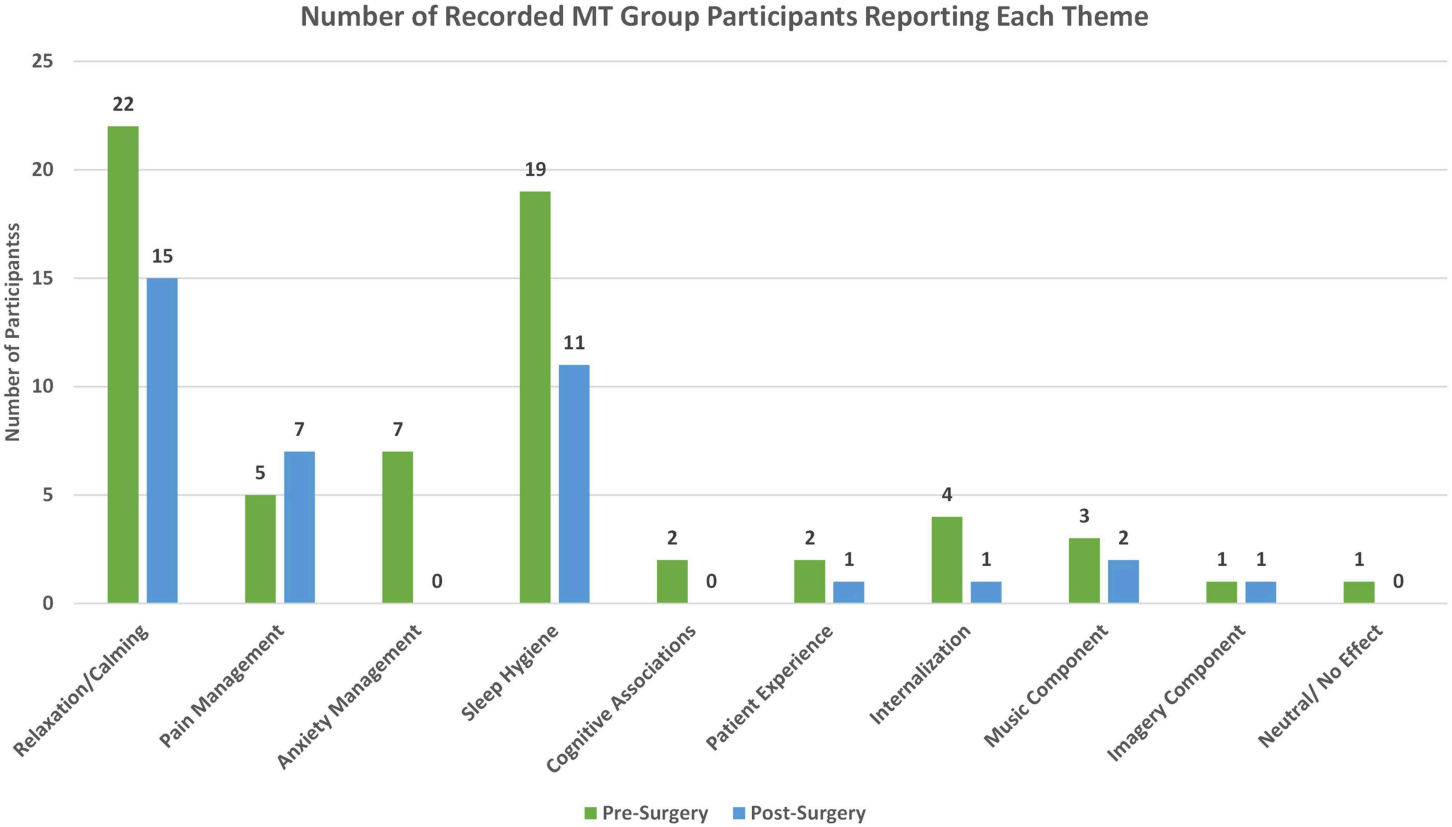
Recorded MT group: pre/postsurgery comparison of themes.

In summary, Figures 1, 2 represent separate results from the Live MT group and the Recorded MT group, with time points tallied as pre- and postsurgery for each.

## Discussion

Following orthopedic surgery, postoperative pain control is a high priority for medical teams. In recognition of the fact that persistent pain may lead to multiple physiological and psychological consequences, including chronic pain, impaired sleep, and increased anxiety, studies have documented the role that music therapy may play in providing non-pharmaceutical relief (46). The purpose of this pilot study was to determine the effects of a conditioned MT intervention on pre- and postoperative pain and anxiety among patients undergoing shoulder surgery. The MT intervention was designed based on a review of the literature and anecdotal clinical evidence over the years prior to applying the approach to these patients. More recent literature continues to support the use of sedative music entrainment (47), autogenic muscle relaxation (40), sedative music with cognitive visualization (48), and opportunity for practice (49).

The results of this longitudinal study yielded informative reactions and perceptions of the patients involved. Although the pilot sample was limited and journal use was inconsistent across participants—thus requiring cautious interpretation—the themes were helpful in identifying patient priorities. Consideration was given to attention effects. Given that the return of journals was essentially even between groups (24 Live MT and 25 Recorded MT), it was concluded that participant willingness to contribute responses may not have been affected by having increased interaction with the music therapist.

Literature reviews speak to the separate effects of music and MT interventions on pain, anxiety, relaxation, and even sleep (50). However, participant journal comments in this study highlighted connections and relationships between pain, anxiety, relaxation, and sleep for patients undergoing shoulder surgery. Participants also mentioned the benefit of practice and repetition, noting the learning and internalization effects, which enhanced their ability to initiate the use of music-based self-help strategies. Multiple themes, both separately and interwoven with one another, emerged, which indicated positive results from participant engagement with the MT intervention.

Overall, given the many spontaneous comments describing MT as being “calming” and “soothing,” the study’s intervention components were largely effective. The intervention was based in a sedative style on guitar which anchored the three different components: the induction song, verbal coaching for autogenic muscle relaxation, and cognitive visualization. Given that the intervention contained multiple active therapeutic components, isolating the specific contribution of each proved to be complex. This was evidenced by most comments referring to the intervention broadly as “the music” or “the CD.” Few references distinguished any specific component as being more effective than the other. More people in the Live MT group than the Recorded MT group commented on the music or “lyrics” rather than the imagery. This was particularly seen during the presurgery time point.

**Figure 3:**
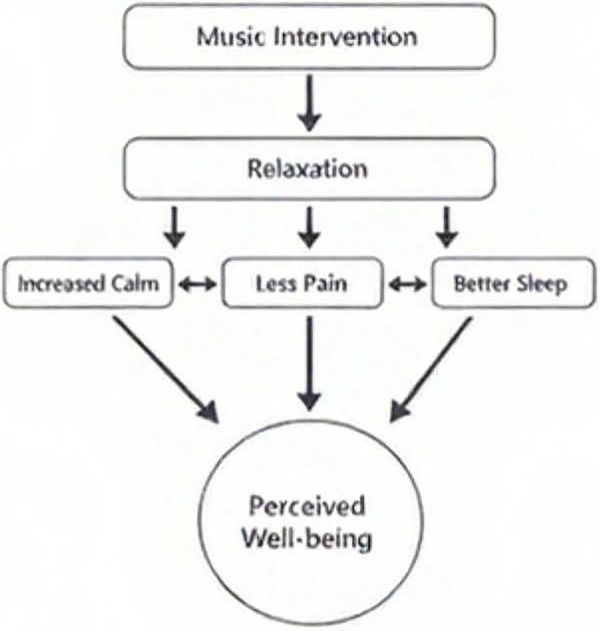
Pathway of music intervention.

Although other studies have reported treatments for pain and anxiety among surgery patients, frequent journal statements in this study indicated that participants' medical conditions also impaired restful sleep. By fostering the initial relaxation response, a progression of effects emerged, as illustrated in Figure 3. For some, the resultant feeling of increased relaxation led to less pain, less anxiety, and better sleep. The interwoven nature of these themes suggests that calming, pain reduction, and sleep are all related to and affected by one another:

Relaxed quickly and slept; shoulder pain when I woke lessened.

Relaxing with music, pain less, keep playing music over and over, fell asleep for 30 min.

Whether through general relaxation and entrainment or active focused shifts of attention, the music intervention was able to help participants calm themselves and relax sufficiently to experience improved pain management and more satisfying sleep.

Participants reported sleep relief in the days leading up to surgery—prior to medical intervention to alleviate the pain of the affected shoulder—and after surgery during recovery and rehabilitation, often linked to physical therapy or “PT” in the comments. Under this category, more people in the Recorded MT group versus the Live MT group reported sleep effects both before and after surgery. This was interesting as both groups had access to the practice CD for use at home. Broadly, however, the calming and relaxing qualities of music were interwoven with the improved ability to sleep, which had a positive effect on participant perceptions of wellbeing.

The opportunity to choose an intervention provided an opportunity for participants to have control over their situation and experience. This, in turn, frequently increased their autonomy and feelings of wellbeing. Journal responses indicated that having the ability to choose an intervention scenario contributed to feelings of agency and control. Whether in the Live or Recorded MT group, participants considered the intervention to be an extension of choice and added support. Journal entries referenced participants perceiving their treatment to be personalized. Statements did not extend to recognition of individualized treatment in the Live MT group, possibly because that group lacked direct experience of the Recorded MT alone for comparison. According to session notes from music therapists at the time of the Live sessions, clinical judgment frequently necessitated in-the-moment adaptation of the intervention protocol to address patient needs. As environmental stimulation varied and other providers conducted preoperative tasks, music therapists adjusted the relaxation scripts to better support participants' immediate physical and mental relaxation needs. Having to deal with specific cases of pain or agitation, it was essential for the music therapists to adopt a different music therapy approach to assist with music-focused engagement through live voice, guitar, and at times, use of live familiar music. This led to additional active relaxation strategies through live music by participating in attentive listening, singing, and discussion. In each instance, music therapists facilitated connections between the participants and the staff preparing patients for surgery or postoperative care. The personalized attention, whether through live interaction or personally chosen recordings, added positive vibes to the participants' perioperative experience.

The participants used the prepared cognitive visualization scenarios and then added personalization from their own lives. Some remembered and focused on certain associated experiences, while others changed their visualization from what was suggested by the therapist and recording to what was more reflective of their life experiences. These responses were noted more in the Live MT group than in the Recorded MT group and more during the presurgery time point than postsurgery. This may have indicated an initial response during earlyexposure which was not repeatedly commented on after participants gained familiarity with the intervention. Certain participants spontaneously donned a more active and creative role in shaping and personalizing their intervention, which may have improved their state of mind and treatment experience. These findings reflect Stuckey and Nobel's (31) thoughts on the relationship between the creative arts and healing. After a review of literature related to the arts and public health, these authors suggested that creativity and imagination are a path to finding a “reservoir of healing.”

Preliminary results from this pilot referenced the initial design question regarding a potential role for conditioning. Having the intervention recordings for use in the days preceding surgery was beneficial from the standpoint of “practice” and “familiarity.” After repeated use, some participants perceived the sedative music and guided intervention as a trigger or cue. Some reported having developed learned associations between the sedative music and perceived feelings of relaxation or calm. A few elaborated further, noting a sense of expectancy that the intervention would assist them during hospitalization. The participants remembered the induction song and sang it to themselves or guided themselves through muscle relaxation when they were aware of the need. Some participants internalized the intervention as a self-help tool for surgery preparation and for managing their anxiousness, stress, and pain.

As researchers did not receive journals from all participants, the potential influence of non-response bias must be considered. Those who did not return journals may have perceived the MT intervention differently or perceived the effects differently from those who supplied data comments. With that being said, the returned journals did include neutral responses, suggestions for change, and indicators of “not liking” the music or components of the intervention. This may indicate that some responding participants felt comfortable providing honest reactions, thereby contributing to a broader scope of perceptions.

In conjunction with this study at the authors' institution, an interdisciplinary pain team was formed to identify risk factors for increased postoperative pain following orthopedic surgery. Following their review, in which anxiety and other psychological and lifestyle conditions were identified as affecting pain outcomes, Armstrong and colleagues developed a pain order set to guide pain management by healthcare providers in their hospital (51). This order set for electronic charting included the ability to directly consult music therapy for evaluation and treatment, which bridged the gap between research and clinical application of multimodal interventions for pain relief and patient care.

## Limitations

Evaluation of this pilot study identified several limitations that could affect transferability and generalization. The non-blinded study design, given the involvement of the music therapist, may have introduced bias, potentially influencing the perceived beneficial effects. Demographic diversity was limited given that participants were enrolled from those presenting for elective shoulder surgery from this single institution. Those eligible to be recruited were predominantly older adults and of generally similar cultural background. Non-English-speaking patients and patients with pre-existing hearing problems were excluded to minimize communication errors in the PACU because of a lack of in-house interpreters at the time of the study. Patients’ personal relationships with music may differ from those of other demographic samples, possibly influencing therapeutic responses.

Use of the journal was inconsistent in several ways. Not all participants returned their journal. Among those who returned it, not all added a comment with a date. Some comments were not dated, making accurate matching of responses to specific points in the surgery timetable difficult. In addition, given the clinical nature of surgical schedules, some participants had more (weeks) or less (days) time between preoperative distribution of the journal and date of surgery. Some participants used the journal only prior to surgery, while others only after surgery, all of which affected the number of pre- and postsurgery comments when mapping use and responses. The control group was not given journals because of the non-blinded, apparent nature of treatment and the journal being used to capture reactions to the MT intervention. However, use of the journal in the control group may have captured patient experience comments regarding pain, anxiety, sleep, standard-of-care interactions with staff, or spontaneous means of preparing for and coping with surgery.

Several methodological aspects were identified for future research. Based on comments such as difficulty “hearing the words,” consideration should be given to determining the best mode of delivery for the treatment protocol at home. Weighing current technology against availability at home and understanding of use by the predominant demographic population is indicated. Also, investigators could include a survey of nursing perceptions to guide generalized clinical application in the hospital setting.

Of particular interest in comparing intervention approaches, consideration may be given to the vocal and verbal elements of the MT treatment and designing the data collection method to capture specific components. Potential exists to move from a verbal-guided visualization provided over sedative guitar accompaniment to an entirely musical approach, extending the induction song with lyrics to support autogenic relaxation and then continuing with songs whose lyrics are based in nature imagery. This approach may be perceived by participants as more similar to hearing familiar, preferred songs than the shift to verbal description in the visualization scripts. Given the identified limitations, the issues of further adaptation of design and means of recruitment should be addressed before the results are transferrable to other surgical populations.

Overall, given the qualitative comments recorded by the participants of this study, the music therapist-designed intervention was effective in supporting a baseline relaxation response. This was evident prior to, immediately after, and during recovery from shoulder replacement surgery. The effects of this music-triggered relaxation included not only increased pain control and anxiety management but also improved sleep and perceptions of a personalized patient experience. Music therapy demonstrated non-pharmaceutical benefits in reducing postoperative pain and associated perioperative anxiety, while also improving sleep and feelings of wellbeing. However, a continued study of approaches and timing is needed to fully optimize patient care.

## Data Availability

The original contributions presented in the study are included in the article/Supplementary Material; further inquiries can be directed to the corresponding author.
