# Supplementary material for: A pilot protocol for conditioning music-cued relaxation responses in patients undergoing total shoulder arthroplasties
Source: Front Pain Res (Lausanne). 2026 Aug 5;7:1872237. doi: 10.3389/fpain.2026.1872237 (PMC13486239; doi:10.3389/fpain.2026.1872237)
Supplement: Supplementary file 1 [file Table1.docx]

**Table 4. Themes and Definitions of Codes**

| **Theme** | **Code** | **Definition** | **No. of Comments / Percent of Total Comments** | **No. of People / Percent of Participants** | | | | | | |
| --- | --- | --- | --- | --- | --- | --- | --- | --- | --- | --- |
|  | | | | | **Live Group** | | | **Recorded Group** | | |
|  |  |  |  | **Total People** | **Total Live** | **Pre- Surgery** | **Post-Surgery** | **Total Recorded** | **Pre-**  **Surgery** | **Post-**  **Surgery** |
| **Theme 1:**  The MT intervention contained qualities and components that were perceived as beneficial for relaxation and calming. | Relaxation and Calming | Perception of feeling calm, relaxed, restful, or decreased muscle tension | 209/65.5% | 47/ 96% | 23/95.8% | 22/ 91.6% | 12/50% | 24/96% | 22/88% | 15/ 60% |
| **Subtheme 1a:**  Pain management | Pain | Perception of physical pain and discomfort | 52/16.3% | 20/40.8% | 10/41.6% | 6/ 25% | 6/25% | 10/40% | 5/20% | 7/28% |
| **Subtheme 1b:** Anxiety Management | Anxiety | Perception of emotional feelings of decreased anxiousness, decreased racing thoughts, peacefulness | 21/6.26% | 21/42.8% | 14/58.3% | 11/45.8% | 3/12.5% | 7/28% | 7/28% | 0 |
| **Subtheme 1c:** Sleep Hygiene | Sleep | Effect on ability to fall asleep, duration, and/or quality of sleep | 113/35.4% | 34/69.3% | 15/62.5% | 14/ 58.3% | 8/ 33.3% | 19/76% | 19/76% | 11/44% |
|  |  |  |  |  |  |  |  |  |  |  |
| **Theme 2:** Participants personalized their visualization experience, which cued a memory and associations of soothing or reflection | Cognitive Associations | Thoughts evoked by the music or cognitive visualization scripts, which brought memories of previous experiences, or interactions, or connections to imagined experiences | 13/4% | 7/14.2% | 5/20.8% | 4/16.6% | 1/ 4.16% | 2/8% | 2/8% | 0 |
|  |  |  |  |  |  |  |  |  |  |  |
| **Theme 3:**  Music therapists were a supportive presence, in addition to the healthcare team. | Patient Experience | Interaction with the healthcare system, aspects of their care, perception of attention and communication | 14/4.38% | 7/14.2% | 4/16.6% | 2/8.3% | 2/8.3% | 3/12% | 2/8% | 1/ 4% |
|  |  |  |  |  |  |  |  |  |  |  |
| **Theme 4:** Through practice, aspects of the music intervention became internalized as a self-help tool. | Internalization | Purposeful practice of the intervention, familiarity, memorization and use, and automatic responses | 17/5.33% | 9/18.3% | 4/ 16.6% | 3/12.5% | 1/ 4.16% | 5/20% | 4/16% | 1/ 4% |
|  |  |  |  |  |  |  |  |  |  |  |
|  | Imagery | References to image, visualization, scene | 4/ 1.25% | 4/ 8.16% | 2/8.3% | 2/8.3% | 0 | 2/ 8% | 1/ 4% | 1/ 4% |
|  | Music | References to music, guitar, singing, lyrics | 25/ 7.83% | 18/ 36.7% | 13/54% | 11/ 44% | 3/ 12.5% | 5/20% | 3/12% | 2/8% |
|  | Neutral / No Effect | References to no effect or benefit, statements not clearly positive or negative. | 5/1.56% | 5/ 10.2% | 4/ 16.6% | 3/ 12.5% | 1/ 4.16% | 1/ 4% | 1/ 4% | 0 |
|  | Unable to Code | Not clear, no informative reference to the study | 6/1.88% | 4/ 8.16% | 1/ 4.16% | 1/ 4.16% | 0 | 3/12% | 2/8% | 1/ 4% |
