# Supplementary material for: A pilot protocol for conditioning music-cued relaxation responses in patients undergoing total shoulder arthroplasties
Source: Front Pain Res (Lausanne). 2026 Aug 5;7:1872237. doi: 10.3389/fpain.2026.1872237 (PMC13486239; doi:10.3389/fpain.2026.1872237)
Supplement: Supplementary file 2 [file Datasheet1.zip › Appendices/Appendix A - Induction Song.pdf]

Appendix A - Induction Song

Listen, Breathe, and Relax: An Induction Song

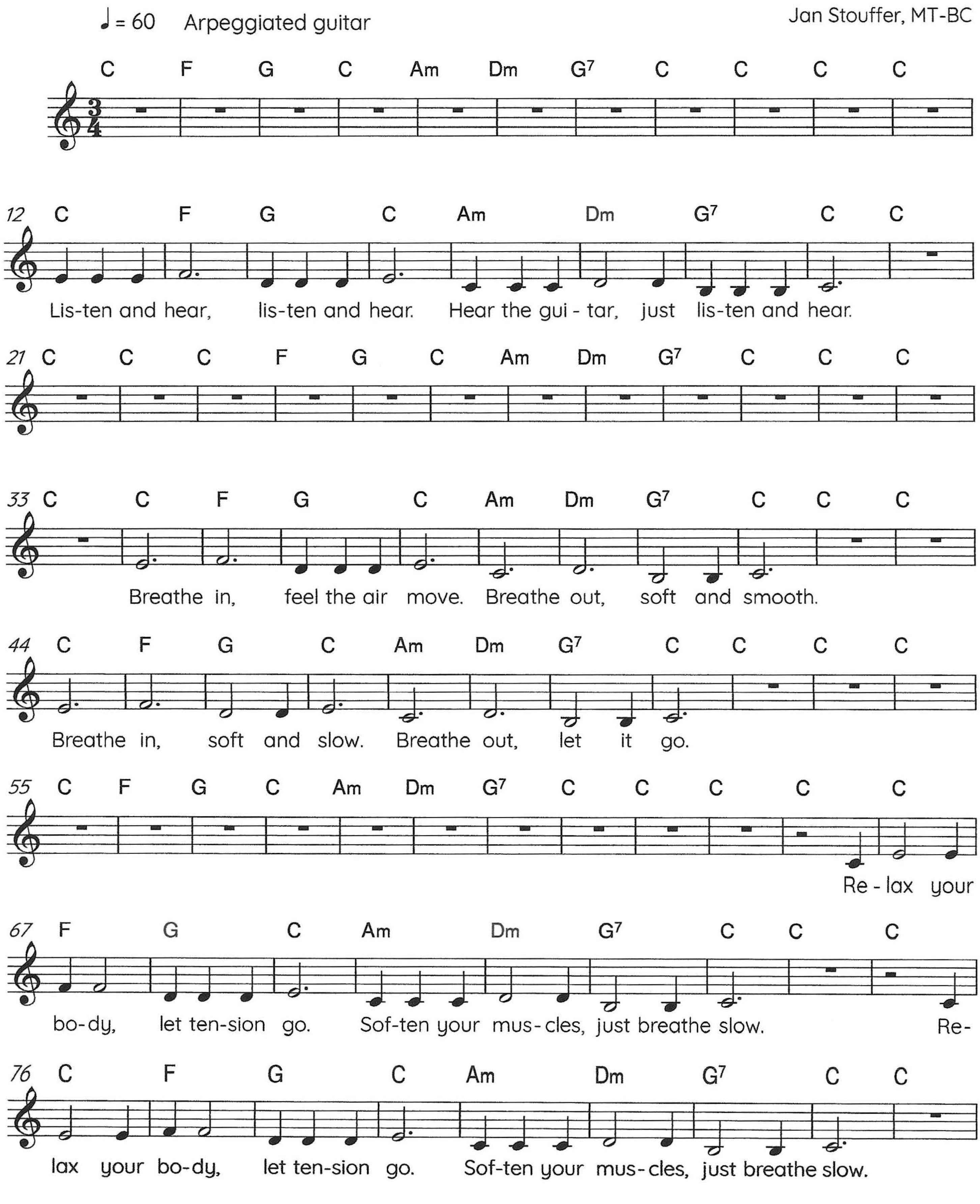

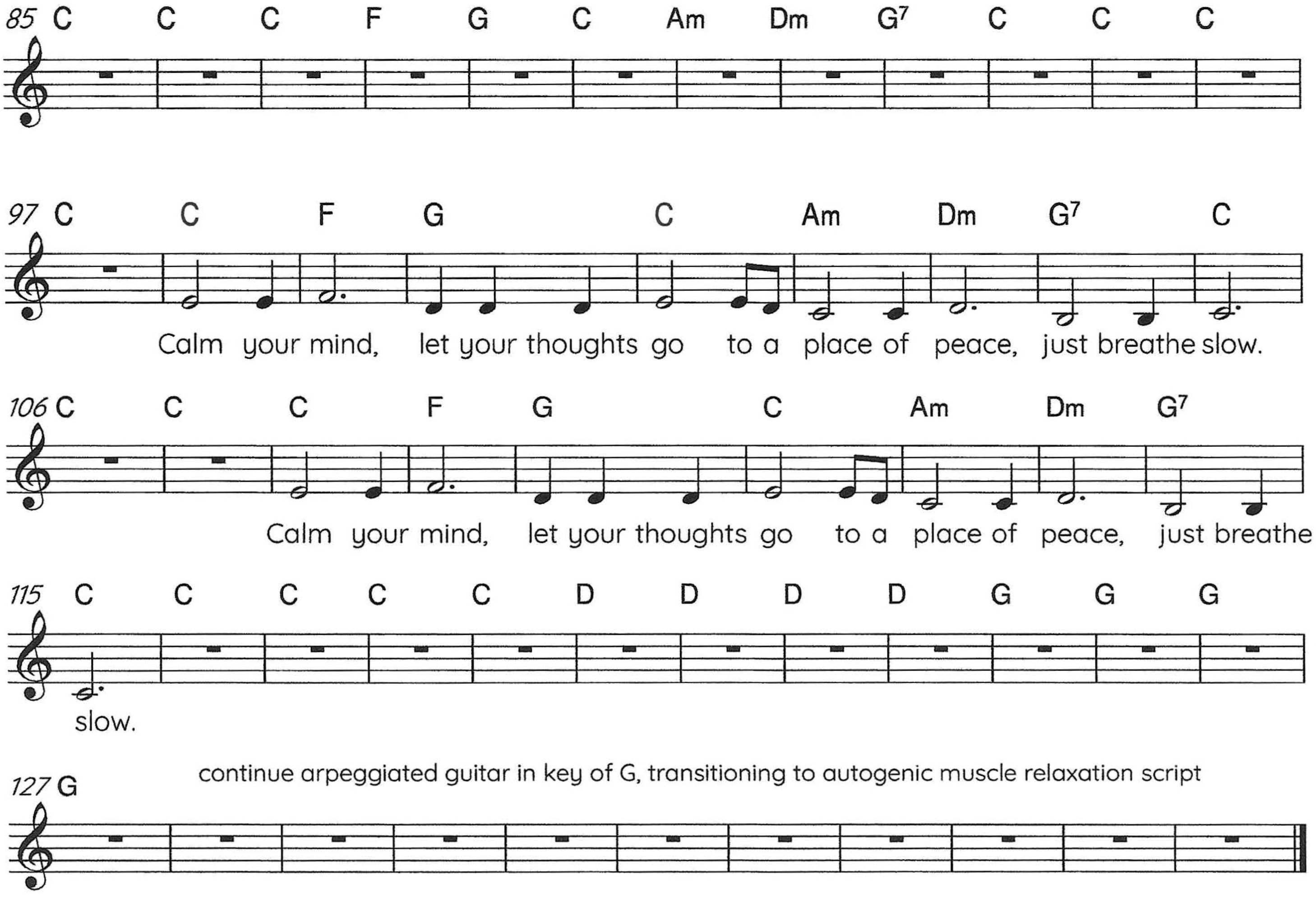
85 C C C F G C Am Dm G7 C C C
97 C C F G C Am Dm G7 C
Calm your mind, let your thoughts go to a place of peace, just breathe slow.
106 C C C F G C Am Dm G7
Calm your mind, let your thoughts go to a place of peace, just breathe
115 C C C C C D D D D G G G
slow.
127 G
continue arpeggiated guitar in key of G, transitioning to autogenic muscle relaxation script
