# Supplementary material for: A pilot protocol for conditioning music-cued relaxation responses in patients undergoing total shoulder arthroplasties
Source: Front Pain Res (Lausanne). 2026 Aug 5;7:1872237. doi: 10.3389/fpain.2026.1872237 (PMC13486239; doi:10.3389/fpain.2026.1872237)
Supplement: Supplementary file 2 [file Datasheet1.zip › Appendices/Appendix B - Autogenic Muscle Relaxation.pdf]

Appendix B
Autogenic Muscle Relaxation Script

[continue sedative guitar picking, Key of G, using progressions including G, Em, C, and D chords]
[use sedative voice tone with appropriate pacing and pauses between phrases to allow response]

As you continue breathing, begin to bring your awareness to your face.

Relax your forehead and your eyes.   Loosen any tension in your jaw.

Allow your neck to simply rest against the bed beneath you. *[resolve to G]*
((with no tightness or pulling)) – {use if sitting in clinic}

Relax your shoulders, let your arms rest gently against your body or the bed.
((or the chair))

Let your breathing be slow and smooth, allowing your chest to relax.

Let your shoulder blades sink into the bed beneath you. *[resolve to G]*
((sink down and soften))

Continue to take nice even breaths,

Bring your awareness to your stomach area,   Relax your stomach,

let the muscles loosen, becoming comfortable and smooth.   *[resolve to G]*

Bring your awareness to your lower back and hips

Allow your lower body to sink into the bed beneath you.   *[resolve to G]*
((sink down to the chair beneath you))

Loosen the muscles in your thighs,   in your calves.

Let any tension that you are still feeling in your body flow down through your legs -
and out through your feet,

Leaving your whole body feeling calm, loose, and relaxed.   *[resolve to G]*

[Continue sedative guitar, transition to cognitive visualization script]
