# Supplementary material for: A pilot protocol for conditioning music-cued relaxation responses in patients undergoing total shoulder arthroplasties
Source: Front Pain Res (Lausanne). 2026 Aug 5;7:1872237. doi: 10.3389/fpain.2026.1872237 (PMC13486239; doi:10.3389/fpain.2026.1872237)
Supplement: Supplementary file 2 [file Datasheet1.zip › Appendices/Appendix C - Cognitive Visualization-Beach.pdf]

Appendix C
Music-Assisted Cognitive Visualization Script - The Beach

[continue sedative guitar picking, Key G, using progressions including G, C2, D, Em, C, Am]
[use sedative voice tone with appropriate pacing and pauses between phrases to allow response]

As you continue your relaxed, even breathing, slowly begin to imagine yourself resting on the beach beside a shallow, protected lagoon. No one is around, just water, smooth sandy beach, and palm trees swaying in the breeze. There are no waves, just the gentle rise and
fall of the water as it laps against the shore. *[resolve to G]*

The water is shallow, a crystal clear blue-green that allows you to see the ocean floor, with just a few small whitecaps cresting the tops of the rises in the tide.

The soft, white sand stretches along the shoreline, dotted by a few, tall palm trees, their fronds gently swaying in the breeze. *[resolve to G]*

The sky is vast, a clear, deep blue that stretches to the horizon. Here and there a few small white clouds drift slowly by.

You smell the fresh, clean air,…. And the mild, salty tang of the sea,….. *[resolve to G]*

The only sounds are the gentle lapping of water as it ebbs and flows towards the shore, and returns back out to sea,….. the quiet, distant call of a bird soaring high overhead,….the breeze softly rustling the palm fronds. *[resolve to G]*

You feel that breeze softly touching your skin. And you feel the gentle warmth of the sun, as it shines down, bathing everything around you in a soft, golden glow. Feel the mild warmth on your skin as you rest on the sand,….warmth, deep and gentle, soaking in to the very heart of you. *[resolve to G]*

Behind your closed eyelids you see the sun's gentle light. Concentrate on the light, for now it is all that you see…. Light, and gentle warmth absorbing into you…. Becoming part of you… and you part of it.
You are warmth and light,…. Flowing with the air around you,…. No effort,…. No thought… *[resolve to G]*

You are slowly and gently being filled with a deep sense of peacefulness and calm, …You feel a sense of light energy, and strength, and an ever-present hope spreading throughout your body, your mind, your heart.

And you realize that you carry these qualities deep inside yourself no matter where you are, no matter what you are doing, and you have them to draw upon whenever you need *[resolve to G]*

As you continue to breathe deeply, you may choose to stay in this place of quiet relaxation, your sheltered lagoon…. Or you can slowly begin to bring this feeling of calm back to where you are now.

You remain calm and relaxed, peaceful, at ease; choosing to rest, or you may slowly open your eyes…

[Complete several final chords in progression, and end on root G note.]
