# Supplementary material for: A pilot protocol for conditioning music-cued relaxation responses in patients undergoing total shoulder arthroplasties
Source: Front Pain Res (Lausanne). 2026 Aug 5;7:1872237. doi: 10.3389/fpain.2026.1872237 (PMC13486239; doi:10.3389/fpain.2026.1872237)
Supplement: Supplementary file 2 [file Datasheet1.zip › Appendices/Appendix D - Cognitive Visualization-Forest.pdf]

Appendix D
Music-Assisted Cognitive Visualization Script – A Forest Stream

[continue sedative guitar picking, Key G, using progressions including G, C2, D, Em, C, Am]
[use sedative voice tone with appropriate pacing and pauses between phrases to allow response]

As you continue your relaxed, even breathing, slowly begin to imagine yourself resting on a large, flat rock,… in the forest, by a stream. All around you are the tall, strong trees, …. With their branches gently moving high overhead, supported by sturdy bark and deep roots. *[resolve to G]*

Low bushes are on the ground around you, some with beautiful mountain flowers,
and everywhere is a carpet of moss and pine needles. High above you the sky is a beautiful, clear blue,….
with just a few white, puffy clouds slowly drifting by. *[resolve to G]*

The smell of pine gives a crisp, clean quality to the air around you. All along the sides of the stream,
the moss-covered rocks line the edge of the water. *[resolve to G]*

You become aware of the stream as it flows beside you, slowly and continuously moving down from its source high up in the mountain, and making its way to places unknown.
The water is clear and clean,… bubbling over the rocks underneath on the stream bed. *[resolve to G]*

You become aware of the gentle feel of the breeze as it touches your skin, as it gently rustles the leaves on the branches of the trees. You feel the gentle warmth of the sunlight as it filters down through the trees, creating a dappled pattern of light and shadow that dances on the forest floor. *[resolve to G]*

You hear the melody of a bird high overhead, singing joyously and effortlessly in its home on the branch of a tree. You hear the sound of the leaves gently moving high overhead, …. the laughter of the stream as it bubbles over the rocks, and you are increasingly aware that all around you is joy, and peace,
and contentment. *[resolve to G]*

As your awareness floats gently back to the water in the stream, you are reminded of its constant, quiet energy as it flows continuously, giving life to everything that it touches,….. Bubbling quickly over the places that are shallow and smooth, moving more slowly over the places that are deep and full of life. *[resolve to G]*

And as you watch the ceaseless flow of the water, you become aware of a place deep inside yourself that seems to be filling with that never-ending source of energy, with the bubbling laughter of joy, with the quiet strength of deep flowing water. You are slowly and gently being filled with a deep sense of peacefulness and calm, …You feel a quiet courage, strength, and an ever-present hope spreading throughout your body, your mind, your heart. And you realize that you carry these qualities deep inside yourself no matter where you are, no matter what you are doing, and you have them to draw upon whenever you need. *[resolve to G]*

As you continue to breathe deeply, you may choose to stay in this place of quiet relaxation, the peacefulness of your mountain stream…. Or you can slowly begin to be aware of where you are now….
You remain calm and relaxed, peaceful, at ease; choosing to rest, or you may slowly open your eyes…

[Complete several final chords in progression, and end on root G note.]
