# Supplementary material for: A pilot protocol for conditioning music-cued relaxation responses in patients undergoing total shoulder arthroplasties
Source: Front Pain Res (Lausanne). 2026 Aug 5;7:1872237. doi: 10.3389/fpain.2026.1872237 (PMC13486239; doi:10.3389/fpain.2026.1872237)
Supplement: Supplementary file 2 [file Datasheet1.zip › Appendices/Appendix E - Cognitive Visualization-Meadow.pdf]

Appendix E

Music-Assisted Cognitive Visualization Script – The Meadow

[continue sedative guitar picking, Key G, using progressions including G, C2, D, Em, C, Am]
[use sedative voice tone with appropriate pacing and pauses between phrases to allow response]

As you continue your relaxed, even breathing, slowly begin to imagine yourself sitting on a blanket on a small bluff above a wide, open meadow. You feel the soft, green grass beneath your feet, and the subtle, delicate warmth of the sun against your skin. *[resolve to G]*

The sky is a clear, deep blue, stretching out around you in all directions.
Spread out below is the open meadow, a clearing that feels like a haven of calm all around you.
The hush of quiet brings peacefulness in this place. *[resolve to G]*

As you sit, gazing across the meadow, you notice the colorful wildflowers spread among the tall green grass. As you breathe in slowly, you smell their soothing scent in the cool, clean air,… The flowers seem to spread a carpet of colors and textures across the floor of the meadow, emphasizing an abundance of joy and life. *[resolve to G]*

Dancing from flower to flower are a few butterflies, gracefully lighting and then moving on again, their wings effortlessly carrying them to what they need, and then to places of rest. *[resolve to G]*

There are trees lining the meadow's edge. You notice the sturdy bark, and branches of the trees, their dark green leaves swaying in the wind. You imagine the deep roots underground, supporting the inner life of the tree. The tallest of the trees seems to touch a few, soft white clouds passing through the sky. *[resolve to G]*

You become aware of the gentle warmth and light of the sun, shining down, bathing the grass and the flowers in a soft, peaceful glow. *[resolve to G]*

As you continue to breathe effortlessly and evenly, you are slowly and gently filled with a deep sense of peacefulness and calm, … You feel a sense of contentment, and strength, and an ever-present hope spreading throughout your body, your mind, your heart.

And you realize that you carry these qualities deep inside yourself no matter where you are, no matter what you are doing, and you have them to draw upon whenever you need *[resolve to G]*

And as you continue to breathe deeply, you may choose to stay in this place of quiet relaxation, the peacefulness of your meadow…. Or you can slowly begin to bring this feeling of calm back to where you are now…. You remain calm and relaxed, peaceful, at ease; choosing to rest, or you may slowly open your eyes…

[Complete several final chords in progression, and end on root G note.]
