# Supplementary material for: A pilot protocol for conditioning music-cued relaxation responses in patients undergoing total shoulder arthroplasties
Source: Front Pain Res (Lausanne). 2026 Aug 5;7:1872237. doi: 10.3389/fpain.2026.1872237 (PMC13486239; doi:10.3389/fpain.2026.1872237)
Supplement: Supplementary file 2 [file Datasheet1.zip › Appendices/Appendix F - Patient Demographic Sheet.pdf]

APPENDIX F
Patient Demographic Sheet

Music Therapy Intervention Groups Only

Patient Initials:__________ **Study ID:______________** **Date:____________**

Imagery Choice: □ Beach □ Forest Stream □ Meadow

If Live Intervention Group:

Required additional MT approaches beyond MAR routine –

Pre-surgery	_____Yes	_____No
Post-surgery	_____Yes	_____No
12-24hr follow up	_____Yes	_____No

If yes, what approach(es) –

_____ Passive listening to live, sedative presentation of patient preferred songs
_____ Active singing along with live, sedative presentation of patient preferred songs
_____ Reminiscence/Association of Meaning/Messages of Coping with songs selected

(Return to Research Coordinator)

For Music Therapists Only (not research data collection):

Typical Music Preferences for Leisure Listening:

For relaxation –

For motivation –

Interests/Leisure Activity:
