# Supplementary material for: A pilot protocol for conditioning music-cued relaxation responses in patients undergoing total shoulder arthroplasties
Source: Front Pain Res (Lausanne). 2026 Aug 5;7:1872237. doi: 10.3389/fpain.2026.1872237 (PMC13486239; doi:10.3389/fpain.2026.1872237)
Supplement: Supplementary file 2 [file Datasheet1.zip › Appendices/Appendix G - Patient Diary.pdf]

APPENDIX G

Relaxation Journal for Surgery Preparation

Patient Initials:__________ Study ID:______________ Date:____________

Instructions to patient: You have been given a recording of a Music-Assisted Relaxation routine which includes calming imagery of your choice. This recording is intended to help you practice relaxation in preparation for your upcoming shoulder surgery. Please use the space below to record dates and times you have used the recording, as well as any comments you have regarding what you feel or think each time you listen.

Date: __________ Time: ________ Comments and Reactions:

Date: __________ Time: ________ Comments and Reactions:

Date: __________ Time: ________ Comments and Reactions:

Date: __________ Time: ________ Comments and Reactions:

Date: __________ Time: ________ Comments and Reactions:

Date: __________ Time: ________ Comments and Reactions:

Date: __________ Time: ________ Comments and Reactions:
